# Prognostic Value of Natriuretic Peptides for All-Cause Mortality, Right Ventricular Failure, Major Adverse Events, and Myocardial Recovery in Advanced Heart Failure Patients Receiving a Left Ventricular Assist Device: A Systematic Review

**DOI:** 10.3389/fcvm.2021.699492

**Published:** 2021-07-07

**Authors:** Eva Janssen, J. Wouter Jukema, Saskia L. M. A. Beeres, Martin J. Schalij, Laurens F. Tops

**Affiliations:** Department of Cardiology, Leiden University Medical Center, Leiden, Netherlands

**Keywords:** left ventricular assist device, circulating biomarkers, natriuretic peptides, adverse events, prognosis

## Abstract

**Aims:** Major adverse event (MAE) rates during left ventricular assist device (LVAD) therapy in advanced heart failure (HF) patients are high, and impair quality of life and survival. Prediction and risk stratification of MAEs in order to improve patient selection and thereby outcome during LVAD therapy is therefore warranted. Circulating natriuretic peptides (NPs) are strong predictors of MAEs and mortality in chronic HF patients. However, whether NPs can identify patients who are at risk of MAEs and mortality or tend toward myocardial recovery after LVAD implantation is unclear. The aim of this systematic review is to analyze the prognostic value of circulating NP levels before LVAD implantation for all-cause mortality, MAEs and myocardial recovery after LVAD implantation.

**Methods and Results:** Electronic databases were searched for studies analyzing circulating NP in adults with advanced HF before LVAD implantation in relation to mortality, MAEs, or myocardial recovery after LVAD implantation. Twenty-four studies published between 2008 and 2021 were included. Follow-up duration ranged from 48 hours to 5 years. Study sample size ranged from 14 to 15,138 patients. Natriuretic peptide levels were not predictive of all-cause mortality. However, NPs were predictive of right ventricular failure (RVF) and MAEs such as ventricular arrhythmias, moderate or severe aortic regurgitation, and all-cause rehospitalization. No relation between NPs and myocardial recovery was found.

**Conclusion:** This systematic review found that NP levels before LVAD implantation are not predictive of all-cause mortality after LVAD implantation. Thus, NP levels may be of limited value in patient selection for LVAD therapy. However, NPs help in risk stratification of MAEs and may be used to identify patients who are at risk for RVF, ventricular arrhythmias, moderate or severe aortic regurgitation, and all-cause rehospitalization after LVAD implantation.

## Introduction

The prognosis of advanced heart failure (HF) is poor, with annual mortality rates over 50%, and limited treatment options (1). Cardiac transplantation is the most effective treatment, although its availability is limited due to insufficient number of donor organs and strict eligibility criteria. Left ventricular assist devices (LVADs) are an alternative treatment option through mechanical unloading of the failing left ventricle (LV). To date, LVAD therapy is increasingly used as destination therapy in patients not eligible for transplantation. Patient selection and timing of LVAD implantation is guided by the profiles of the Interagency Registry for Mechanically Assisted Circulatory Support (INTERMACS) classifying patients with advanced heart failure (2).

The most recent INTERMACS report has shown a 1-year survival rate of 79–80% in patients receiving continuous flow (CF)-LVAD therapy (3, 4). Major adverse event (MAE) and rehospitalization rates are high, and impair quality of life and survival (4, 5). These MAEs include neurologic event (defined as stroke or transient ischemic attack), gastrointestinal bleeding, major infection, and right heart failure (RVF) occurring 13–20, 20–25, 40–43, and 29–38% at 1 year after CF-LVAD implantation, respectively (4). All-cause rehospitalization rates were 21–23% (4). Device explantation for LV myocardial recovery is rare with 3.1% at 3 years, and <5% at 5 years follow-up (3, 6). It would be beneficial to identify patients prone to myocardial recovery, and consider adjustments of their pharmacological treatment (7). To identify patients at risk of MAEs and early mortality would be of great importance. Treatment options for MAEs are limited and often ineffective, having corresponding high mortality rates. Thus, prediction and risk stratification of MAEs before LVAD implantation is warranted in order to improve patient selection, and thereby outcome of LVAD therapy. Measurement of circulating biomarkers such as natriuretic peptides (NPs) may help in risk stratification.

**Figure 1:**
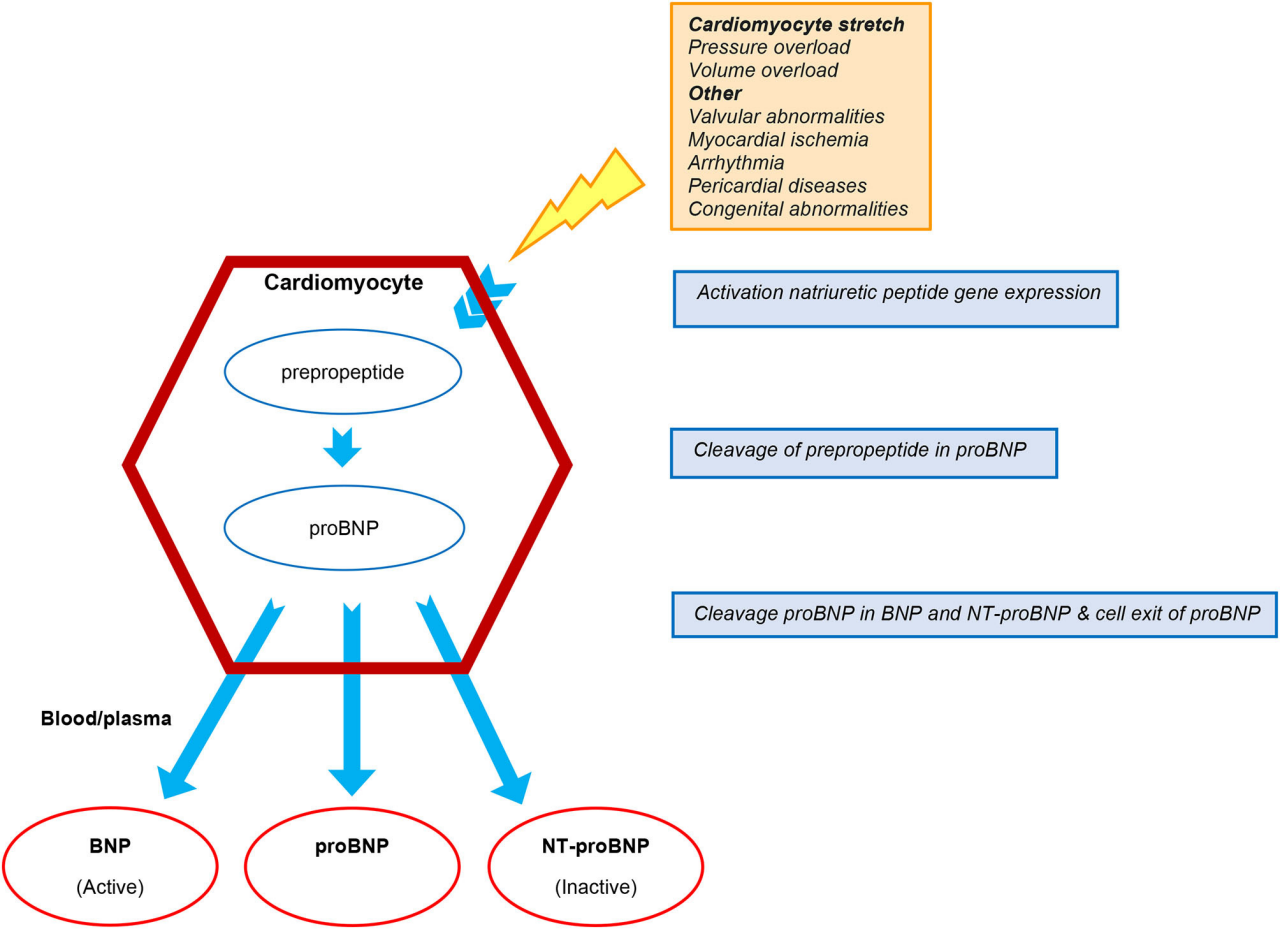
Production and cleavage of proBNP. BNP, B-type natriuretic peptide; NT-proBNP, N-terminal pro-BNP.

Three subtypes of NP are known; atrial natriuretic peptide (ANP), B-type natriuretic peptide (BNP), and C-type natriuretic peptide (CNP) (8, 9). Natriuretic peptides mainly reflect the hemodynamic burden of the failing heart, and are regulated by volume overload and neuro-hormonal stimulation. Prehormone pro-BNP is released by cardiomyocytes in reaction to mechanical stretch and myocardial ischemia. Upon secretion into the circulation it is cleaved in biologically active BNP and its inactive remnant N-terminal proBNP (NT-proBNP) (Figure 1) (8–12). Levels of NP are influenced by various factors including age, gender, comorbidities, renal function, pulmonary disease, and obesity (13–15). Heart failure medication, including beta-blockers, diuretics, and inotropes affect NP levels, reflecting the improvement in hemodynamic state induced by these therapies. The novel HF drug sacubitril/valsartan influences BNP and NT-proBNP levels differently, in particular during the first 8–10 weeks after initiation. Whereas, the use of sacubitril/valsartan, a neprilysin inhibitor, may increase the circulating levels of BNP, it does not affect the circulating levels of NT-proBNP since the latter is not a substrate of neprilysin inhibition. Nonetheless, both BNP and NT-proBNP have prognostic value during treatment with sacubitril/valsartan (8, 16). Finally, it has been demonstrated that a large percentage of measured circulating BNP or NT-proBNP is in fact their prehormone proBNP. Therefore, BNP, NT-proBNP, and proBNP measurements from different assays are not reliably comparable due to their differences in cross-reactivity (17, 18).

In the American College of Cardiology/American Heart Association guideline, BNP and NT-proBNP have a class IA recommendation to establish disease severity and prognosis of patients with chronic HF (2). Hutfless et al. showed that preoperative BNP levels are strong predictors of postoperative need for intra-aortic balloon pump, longer postoperative hospital stay, and higher 1-year mortality in patients undergoing open heart surgery (19). Furthermore, the prognostic value of NP levels related to all-cause mortality, adverse events, and rehospitalization in chronic HF patients has been well-established (20–24).

Whether preoperative NP levels can improve patient selection for LVAD therapy by identifying patients who are at risk for early all-cause mortality, right ventricular failure (RVF), or MAEs, and can identify patients who tend toward myocardial recovery after LVAD implantation, is not yet systematically evaluated. In this review, we sought to systematically evaluate the prognostic value of circulating NP levels in advanced HF patients before LVAD implantation for all-cause mortality, RVF, MAEs including rehospitalization, and myocardial recovery after successful LVAD implantation.

## Methods

This systematic review is written in accordance with the Preferred Reporting Items for Systematic Reviews and Meta-analyses (PRISMA) guideline (Supplementary Material 1). Since individual patient data are not included, institutional review board approval was not required.

### Literature Search and Selection

Seven electronic databases were searched: MEDLINE, Web of Science Core Collection, Cochrane Reviews, Cochrane Trials, PubMed, Factiva, and Embase. The following (MeSH) terms were used: “left ventricular assist device,” “ventricular assist device,” “mechanical circulatory support,” “biomarkers,” “natriuretic peptide,” “B-type natriuretic peptide,” “brain natriuretic peptide,” “pro B-type natriuretic peptide,” “pro brain natriuretic peptide,” “NT-pro B-type natriuretic peptide,” “N-terminal pro B-type natriuretic peptide,” and “N-terminal pro brain natriuretic peptide.” The search was restricted to human studies published in English up to January 1st, 2021. Study selection criteria were predefined as described in Table 1.

**Table 1:** Inclusion–and exclusion criteria.

**Inclusion**
Patient population: humans > 18 years with advanced HF who will receive left ventricular assist device therapy
Outcome(s): all-cause mortality, right ventricular failure, major adverse events, myocardial recovery
Prognostic factor: circulating natriuretic peptide levels measured before LVAD implantation
Language: English
**Exclusion**
Reviews, editorials, case reports, abstracts

The authors of this manuscript screened the titles and abstracts of all studies retrieved from the literature search. Potentially relevant studies, or studies whose relevance could not be ascertained based on the abstract, were screened full text. A single assessor screened each article full text for inclusion. Corresponding authors were contacted to obtain full data not covered in the publication.

### Data Collection

Extracted data included details of the patient population, etiology of HF, type of VAD, device strategy, timing of blood sampling for NPs measurement, type of NPs, cut-off points of NPs (when available), type of statistical analysis, adjusted variables for multivariate analysis, and duration of follow-up. The outcomes all-cause mortality, RVF, MAEs (including all-cause planned and unplanned rehospitalization) and myocardial recovery, and their describing definitions were extracted. Major adverse events were defined according to the “2020 Updated definitions of adverse events for trials and registries of mechanical circulatory support” (25). In the current review, the following MAEs were included: ventricular arrhythmia (VA), aortic regurgitation (AR), “combined adverse events” (including episode of VA, HF, chest pain, bleeding, infection, thrombosis, pump-related problems, biliary disfunction, elective procedures), complicated postoperative stay, and all-cause rehospitalization.

### Study Quality

The Newcastle–Ottawa Scale (NOS) for observational cohorts was used to assess the quality of all included studies (26). The NOS score was then converted into Agency for Healthcare Research and Quality standards (AHRQ); good, fair, and poor (27).

## Results

### Literature Search

**Figure 2:**
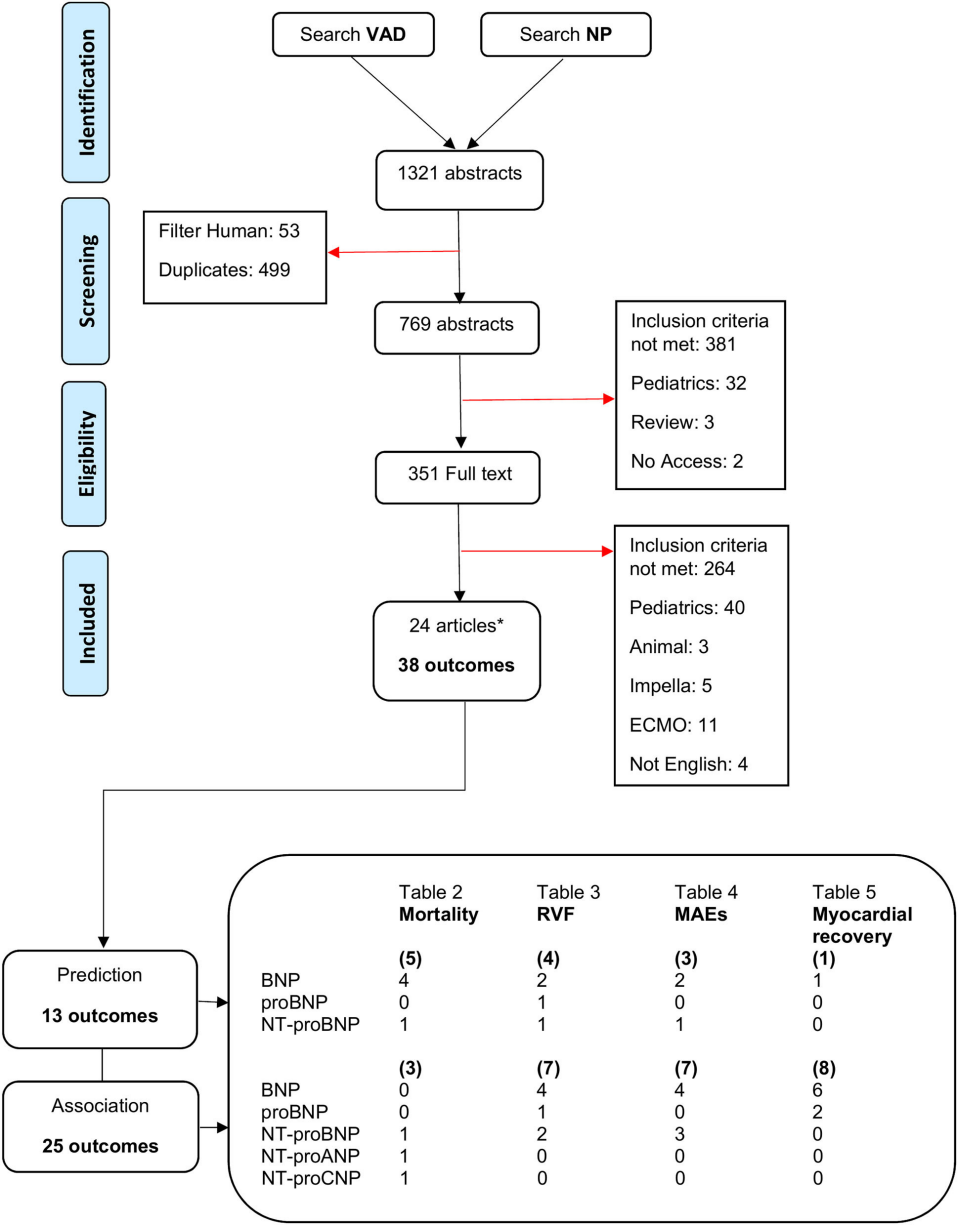
PRISMA flow diagram for literature search and study selection process. *Several articles contain multiple circulating NP or outcomes. ANP, atrial natriuretic peptide; BNP, B-type natriuretic peptide; CNP, C-type natriuretic peptide; ECMO, extracorporeal membrane oxygenation; MAEs, major adverse events; NP, natriuretic peptide; NT-proBNP, N-terminal pro-BNP; RVF, right ventricular failure; VAD, ventricular assist device.

The literature search and selection process is presented in the PRISMA flow diagram in Figure 2. The literature search retrieved 1,321 citations from the seven electronic databases. After duplicates were removed, 769 citations went through title and abstract screening, of which 351 articles were screened full text. A total of 745 citations were excluded; the majority did not meet the inclusion criteria regarding advanced HF, reporting any kind of endpoint, receiving LVAD therapy, or measurement of circulating NPs prior to device implantation. Eventually, 24 articles passed full text screening and were included in this review.

### Study Characteristics

The included studies were published between 2008 and 2020, and were from countries in Europe, the United States of America, and Japan. Twenty-three of the included studies were retrospective cohort studies. The 24 studies were fairly heterogenous reporting on multiple subtypes of NP, and various and multiple outcomes. This resulted in a total of 38 outcomes in all studies, where predictive relations were studied in 13 and associative relations in 25 (Figure 2). Follow-up duration ranged from 48 hours up to 5 years after LVAD implantation. Study sample sizes ranged from 14 to 15,138 patients. Natriuretic peptides were extracted from various materials (blood or plasma), measured with different assays, and presented in diverse measuring units. The upper cut off levels for normal NP levels varied from one study to the other. It should be pointed out that NP levels, unless log transformed, are non-normal distributed. Nevertheless, several studies included in this review chose to report NP levels non-log transformed. Descriptive statistic, mean ± standard deviation (SD), was used for log transformed NP measurements. Median and (interquartile) range was used for non-log transformed NP measurements. Groups were compared using various analyses depending on how continuous variables were expressed and how many groups were compared. Univariate and multivariate Cox regression was used for survival analyses expressed in hazard ratio. Univariate and multivariate logistic regression was used to estimate the strength of the effect of NPs expressed in odds ratio. The quality assessed by the NOS was “good” in all studies (Supplementary Material 2).

### Study Results

The 24 studies assessing the relation between NP levels before LVAD implantation and all-cause mortality, RVF, MAEs, and myocardial recovery after implantation are summarized in Tables 2–5.

**Table 2:** Baseline characteristics and outcomes of included studies for all-cause mortality.

**References**	**NP**	**Timing NP** **days**	**Design** ***N***	**LVAD/CF *N (%)***	**DT/BTT** ***N (%)***	**Male gender** ***N (%)***	**Age** **years**	**ICM** ***N (%)***	**FU days**	**Definition outcome** **mortality**	**Statistics**	**Relation NPs—mortality**
**Predictive relation**												
Papathanasiou et al. (28)	BNP- Log pg/ml	2	RS 103	103 (100) 103 (100)	69 (67) 34 (33)	82 (80)	59 (11)^†^	56 (54)	180	All-cause mortality	HR, 95%CI HR, 95%CI	1.27 (0.94–1.71) *p =* 0.12 1.35 (0.92–1.96)^‡^ *p =* 0.12
										*All-cause mortality or rehospitalization*	HR, 95%CI HR, 95%CI	*1.10 (0.87–1.37) p = 0.45* *1.00 (0.76–1.33)*^‡^ *p = 0.98*
Sato et al. (29)	BNP- Log pg/ml	1	RS 83	83 (100) 18 (22)	83 (100) 0 (0)	63 (76)	39 ± 12	3 (4)	90	All-cause mortality	HR, 95%CI	1.00 (0.99–1.00) *p =* 0.673
Yoshioka et al. (30)	BNP pg/ml	-	RS 41	41 (100) 6 (15)	0 (0) 41 (100)	29 (71)	39 ± 2	3 (7)	90	All-cause mortality	OR, 95%CI	1.00 (0.99–1.00) *p =* 0.246
Shiga et al. (31)	BNP pg/ml	-	RS 47	47 (100) 0 (0)	0 (0) 47 (100)	35 (75)	39 ± 15	12 (26)	730	All-cause mortality	OR, 95%CI	1.000 (1.000–1.001) *p =* 0.576
	BNP ≥1,000 pg/ml										OR, 95%CI	1.143 (0.382–3.421) *p =* 0.812
Topilsky et al. (32)	NT-proBNP per 100 increase pg/ml	1	R- 83	83 (100) 83 (100)	56 (67) 27 (23)	81 (98)	63 ± 12	45 (54)	30	Mortality	OR, 95%CI	1.03 (1.01–1.06) ***p=*** **0.003**
**Associative relation**												**Survivors vs. non-survivors Median (range)**
Cabiati et al. (33)	NT-proBNP pg/ml	Hospital admission	RS 17	17 (100) 17 (100)	- -	16 (94)	51 (47–63)^†^	5 (28)	28	All-cause mortality	*χ2* test	986.10 vs. 5721.00 ***p=*** **0.028**
	NT-proANP nmol/l	Hospital admission	RS 17	17 (100) 17 (100)	- -	16 (94)	51 (47–63)^†^	5 (28)	28	All-cause mortality	*χ2* test	8.04 vs. 11.20 *p =* 0.832
	NT-proCNP pg/ml	Hospital admission	RS 17	17 (100) 17 (100)	- -	16 (94)	51 (47–63)^†^	5 (28)	28	All-cause mortality	*χ2* test	85.92 vs. 52.07 *p =* 0.322

*Age in years is described in Mean ± SD, except for ^†^Median (range).*

*^‡^Adjusted for age, male gender, DT, EF, creatinine, CKD, ECLS, IABP, invasive ventilation, inotropes.*

*BNP, B-type natriuretic peptide; BTT, bridge to transplant; CKD, chronic kidney disease; CF, continuous flow; CI confidence interval; DT, destination therapy; ECLS, Extra Corporeal Life Support; EF, ejection fraction; FU, follow up; HR, hazards ratio; IABP, intra-aortic balloon pump; ICM, ischemic cardiomyopathy; Log, log transformed, LVAD, left ventricular assist device; NP, natriuretic peptide; NT-proBNP; N-terminal pro-B-type NP; N, number; OR, odds ratio; R, retrospective cohort; S, single center; SD, standard deviation; vs., versus.*

*The bold p-values indicate statistical significance*.

**Table 3:** Baseline characteristics and outcomes of included studies for right ventricular failure.

**References**	**NP**	**Timing NP** **days**	**Design** ***N***	**LVAD/CF** ***N (%)***	**DT/BTT** ***N (%)***	**Male gender** ***N (%)***	**Age years**	**ICM** ***N (%)***	**FU** ***Days***	**Definition outcome** **RVF**	**Statistics**	**Relation NPs—RVF**
**Predictive relation**												
Shiga et al. (34)	BNP- Log pg/ml	-	RS 79	79 (100) 20 (25)	0 (0) 79 (100)	58 (73)	39 ± 14	14 (18)	PO	ECMO, or need for RVAD	OR, 95%CI	1.001 (1.000–1.001) ***p=*** **0.043**
	BNP ≥1,200 pg/ml										OR, 95%CI	8.409 (0.922–76.73) *p =* 0.059
Kato et al. (35)	BNP >1,232 ng/ml	≤ 5	RS 61	61 (100) 30 (49)	- -	52 (85)	54 ± 13	25 (41)	2–14	NO inhalation >48 h, and/or restarting/ inotropic support >14 days, or need for RVAD	OR, 95%CI	1.021 (1.000–1.042) ***p=*** **0.035**
											OR, 95%CI	1.021(1.000–1.027)^‡^ ***p=*** **0.0357**
Loghmanpour et al. (36)	proBNP -	-	RM 10909	10909 (100) 10909 (100)	3811 (35) 6901 (63)	8,606 (78)	(50–69)^†^	4,466 (41)	2–14	Pharmacological management of RVF/PVR, or need for RVAD	Bayesian model; 176 variables	**6th most powerful predictor out of 176**
Potapov et al. (37)	NT-proBNP >10,000 pg/ml	1	R- 54	54 (100) 37 (69)	- -	49 (91)	52 (32–69)^†^	6 (11)	2	In absence of cardiac tamponade 2 criteria, MAP ≤ 55 mmHg, CVP ≥16 mmHg, mixed VS ≤ 55%, CI <2 L/min/m^2^, IS >20 h, or need for RVAD	OR, 95%CI	1 (1–1.002) *p =* 0.1
**Associative relation**												**RVF vs. no RVF Mean** **±** **SD**
Kapelios et al. (38)	BNP pg/ml	-	RS 20	20 (100) 20 (100)	20 (100) 0 (100)	19 (95)	54 ± 10	12 (60)	1,241 ± 694*	>1 year: inotrope: iv or inhaled vasodilator (>7days), tqo of the four criteria; CVP >18 mmHg or mean RAP >18 mmHg, CI <2.3 L/min/m^2^, ascites/ peripheral edema, CVP>, or need for RVAD	Paired t-test	1,819 ± 1,492 vs. 1,359 ± 1,611 *p =* 0.52
Kato et al. (35)	BNP pg/ml	≤ 5	RS 61	61 (100) 30 (49)	- -	52 (85)	54 ± 13	25 (41)	2–14	NO inhalation >48 h, and/or restarting/ inotropic support >14 days, or need for RVAD	Paired t-test	1895.1 ± 1551.1 vs. 1250.5 ± 1045.2 *p =* 0.0572
Shiga et al. (34)	BNP- Log pg/ml	-	RS 79	79 (100) 20 (25)	0 (0) 79 (100)	58 (73)	39 ± 14	14 (18)	PO	ECMO, or need for RVAD	*χ2* test	7.55 ± 0.60 vs. 6.76 ± 0.90 ***p=*** **0.041**
Deswarte et al. (39)	BNP pg/ml	-	RM 14	14 (100) 14 (100)	14 (100) 0 (0)	-	63 (37–69)^†^	7 (50)	30	Inotropic support ≤ 14days, death caused by RVF, or need for RVAD	Mann-Whitney *U*-test	1,792 (992–8,500) vs. 1,710 (701–3,643)^§^ *p >* 0.05^#^
Pettinari et al. (40)	proBNP -	-	R- 59	59 (100) 59 (100)	4 (7) 55 (93)	53 (90)	48 ± 15	31 (52)	PO	Need for RVAD	Mann-Whitney *U*-test	11,034 ± 9,620 vs. 4,667 ± 3,082 *p =* 0.06
Hennig et al. (41)	NT-proBNP pg/ml	1	RS 40	40 (100) 22 (55)	0 (0) 40 (100)	38 (95)	54 ± 13	-	2	In absence of cardiac tamponade two criteria: MAP ≤ 55 mmHg, CVP ≥16 mmHg, mixed VS ≤ 55%, CI <2 L/min/m^2^, IS >20 h; or need for RVAD	Mann-Whitney *U*-test or *χ2* test	17,174 vs. 6,322^†^ ***p=*** **0.032**
Potapov et al. (37)	NT-proBNP pg/ml	1	R- 54	54 (100) 37 (69)	– –	49 (91)	52 (32–69)^†^	6 (11)	2	In absence of cardiac tamponade two criteria: MAP ≤ 55 mmHg, CVP ≥16 mmHg, mixed VS ≤ 55%, CI <2 L/min/m^2^, IS >20 h; or need for RVAD	Mann-Whitney *U*-test or t-test	13,026 (8,800–17,566) vs. 4,699 (925–10,433)^†^ ***p=*** **0.003**

*Age in years is described in Mean ± SD, except for ^†^ Median (range).*

*^‡^Adjusted for PAP, RAP, RVSWI, tBili, RV FAC, serum OPN.*

^*^Mean ± SD.

*^§^Mean (interquartile range).*

*^#^Exact p-value unknown.*

*BNP, B-type natriuretic peptide; BTT, bridge to transplant; CF, continuous flow; CI, cardiac index; CI confidence interval; CVP, central venous pressure; DT, destination therapy; ECMO, Extracorporeal membrane oxygenation; FU, follow up; h, hours; HR, hazards ratio; ICM, ischemic cardiomyopathy; IS, inotropic support; Log, log transformed, LVAD, left ventricular assist device; M, multicenter; MAP, mean arterial pressure; N, number; NO, nitric oxide; NP, natriuretic peptide; NT-proBNP; N-terminal pro-B-type NP; OPN, osteopontin; OR, odds ratio; PAP, pulmonary artery pressure; PO, postoperative; PVR, pulmonary vascular resistance; R, retrospective cohort; RVAD, right ventricular assist device; RVF, right ventricular failure; RV FAC, right ventricular fractional area change; RVSWI, right ventricular stroke work index; S, single center; SD, standard deviation; tBili, total bilirubin concentration; vs., versus; VS, venous saturation.*

*The bold p-values indicate statistical significance*.

**Table 4:** Baseline characteristics and outcomes of included studies for major adverse events.

**References**	**NP**	**Timing NP** **days**	**Design** ***N***	**LVAD/CF** ***N (%)***	**DT/BTT** ***N (%)***	**Male** **gender** ***N (%)***	**Age** **years**	**ICM** ***N (%)***	**FU** ***Days***	**Definition outcome** **MAEs**	**Statistics**	**Relation NPs—MAEs**
**Predictive Relation**												
Truby et al. (42)	BNP >500 ng/l	-	RM 10603	10,279 (97) 10,603 (100)	4,474 (42) 6,047 (57)	8,246 (78)	>60 (45)*	4,738 (45)	730	Moderate or severe AR	HR, 95%CI	1.48 (1.23–1.77) ***p<*** **0.001**
Hellman et al. (43)	BNP max. pg/ml	122	RS 74	74 (100) 74 (100)	- -	49 (66)	56	30 (41)	15	VA: VF, VT, or asymptomatic NSVT	OR, 95%CI	1.5–5.1^‡^ ***p=*** **0.0008**
Hasin et al. (44)	NT-proBNP per 1,000 increase pg/ml	Hospital admission	RS 88	115 (100) 115 (100)	73 (63) 42 (37)	96 (83)	62 (53–69)^†^	56 (49)	511 ± 329^#^	**Less** rehospitalization: Cardiac (VA, HF, chest pain), bleeding, infection, thrombosis, pump related, biliary, elective, other	HR, 95%CI HR, 95%CI	0.98 (0.96–0.99) ***p=*** **0.022** 0.98 (0.96–1.00)^§^ ***p=*** **0.022**
		**Associative Relation**										**Outcome vs. no outcome Median (range)**
Truby et al. (42)	BNP ng/l	-	RM 10603	10,279 (97) 10,603 (100)	4,474 (42) 6047 (57)	8,246 (78)	>60 (45)*	4,738 (45)	730	Moderate or severe AR	Kruskal-Wallis test	915 (489–1783) vs. 756 (382–1421) ***p=*** **0.001**
Hegarova et al. (45)	BNP ng/l	1	PS 136	136 (100) 136 (100)	0 (0) 136 (100)	121 (89)	51 (23–72)^†^	54 (40)	298 (159–456)^†^	**Less** adverse events; HF, infection, pump thrombosis	Mann-Whitney *U*-test	1440.4 vs. 2405.5^#^ ***p=*** **0.001**
	BNP ng/l	1	PS 59	59 (100) 59 (100)	0 (0) 59 (100)	51 (86)	51 (23–72)^†^	23 (39)	298 (159–456)^†^	Rehospitalization	Mann-Whitney *U*-test	1118.7 vs. 1762.1^#^ *p =* 0.056
Hellman et al. (43)	BNP pg/ml	122	RS 74	74 (100) 74 (100)	- -	49 (66)	56	30 (41)	15	VA: VF, VT, or asymptomatic NSVT	Mann-Whitney *U*-test	2,373 vs. 1,309 ***p=*** **0.0016**
Hasin et al. (46)	NT-proBNP pg/ml	Hospital admission	RS 72	72 (100) 72 (100)	- 21 (29)	63 (87)	63 (53–69)^†^	36 (50)	14	Complicated postoperative stay: IC > 5days, ventilator support > 2days, total hospital stay >14 days	Mann-Whitney *U*-test	4,786 (2,232–13,790) vs. 3,199 (869–11,803) *p =* 0.224
	NT-proBNP Log pg/ml											1.14 (0.46–2.16) vs. 1.09 (0.32–3.30)^‡‡^ *p =* 0.725
	NT-proBNP pg/ml	1	RS 72	72 (100) 72 (100)	- 21 (29)	63 (87)	63 (53–69)^†^	36 (50)	14	Complicated postoperative stay: IC >5 days, ventilator support >2 days, total hospital stay >14 days	Mann-Whitney *U*-test	3,446 (1,801–8,101) vs. 3,431 (932–5,113) *p =* 0.187
	NT-proBNP Log pg/ml											1.11 (0.52–2.72) vs. 0.98 (0.49–2.16)^‡‡^ *p =* 0.410
Hasin et al. (44)	NT-proBNP Per 1,000 expressed pg/ml	Hospital admission	RS 115^‡^	115 (100) 115 (100)	73 (63) 42 (37)	96 (83)	62 (53–69)^†^	56 (49)	511 ± 329^#^	**Less** rehospitalization: Cardiac (VA, HF, chest pain), bleeding, infection, thrombosis, pump related, biliary, elective, other	Wilcoxon signed rank test	4.3 (2.4–8.3) vs. 4.7 (3.0–8.1) *p =* 0.022

Age in years is described in Mean ± SD, except for ^*^Percentage with age > 60 years and ^†^Median (range).

*^‡^Adjusted for VT in the past, ICD, CAD, and age.*

*^§^Adjusted for referral zone, neighboring states, and hemoglobin.*

*^#^Mean ± SD.*

*Adjusted for age, gender, and glomerular filtration rate.*

*AR, aortic regurgitation; BNP, B-type natriuretic peptide; BTT, bridge to transplant; CAD, coronary artery disease; CF, continuous flow; CI confidence interval; DT, destination therapy; FU, follow up; HF, heart failure; HR, hazards ratio; IC, intensive care; ICD, internal cardiac defibrillator; ICM, ischemic cardiomyopathy; Log, log transformed, LVAD, left ventricular assist device; M, multicenter; MAEs, major adverse events; Max, maximal level measured; NP, natriuretic peptide; NT-proBNP; N-terminal pro-B-type NP; N, number; OR, odds ratio; P, prospective cohort; R, retrospective cohort; S, single center; SD, standard deviation; VA, ventricular arrhythmia; VF, ventricular fibrillation; (NS)VT, non-sustained ventricular tachycardia; vs., versus.*

*The bold p-values indicate statistical significance*.

**Table 5:** Baseline characteristics and outcomes of included studies for left ventricular (LV) myocardial recovery.

**References**	**NP**	**Timing NP Days**	**Design *N***	**LVAD/CF *N (%)***	**DT/BTT *N (%)***	**Male gender *N (%)***	**Age *Years***	**ICM *N (%)***	**FU *Days***	**Definition outcome Myocardial recovery**	**Statistics**	**Relation NPs—Myocardial recovery**
**Predictive relation**												
Imamura et al. (47)	BNP- Log pg/ml	1	RS 27	27 (100) 22 (81)	0 (0) 27 (100)	21 (78)	35 ± 14	0 (0)	183	LVRR (EF ≥35%)	OR, 95%CI	0.753 (0.021–27.17) *p =* 0.877
		**Associative relation**										**Myocardial recovery vs. no myocardial recovery Mean** **±** **SD**
Topkara et al. (6)	BNP pg/ml	-	RM 13454	13,454 (100) 13,454 (100)	5,257 (39) 3,714 (28)	10,567 (79)	51.5 ± 13	6,241 (46)	1,096	Device explant vs. no device explant	Unpaired *t*-test	926.7 ± 860.9 vs. 1169.9 ± 1097.6 ***p=*** **0.024**
			RM 8805	8,805 (100) 8,805 (100)	351 (46)	6,918 (79)	57.1 ± 14	3942 (29)	1,096	LVRR (EF ≥40%) vs. no LVRR (EF <30%)	Unpaired *t*-test	1240.9 ± 149.5 vs. 1157.4 ± 1086.1 *p =* 0.199
	proBNP pg/ml	-	RM 13454	13,454 (100) 13,454 (100)	241 (32) 5,257(39)	10,567 (79)	51.5 ± 13	6241 (46)	1,096	Device explant vs. no device explant	Unpaired *t*-test	4642.4 ± 7323.3 vs. 6787.2 ± 7887.3 *p =* 0.153
			RM 8805	8,805 (100) 8,805 (100)	3,714 (28) 351 (46) 241 (32)	6,918 (79)	57.1 ± 14	3942 (29)	1,096	LVRR (EF ≥40%) vs. no LVRR (EF <30%)	Unpaired *t*-test	9880.8 ± 11664.4 vs. 6617.3 ± 7737.5 ***p=*** **0.003**
Wever-Pinzon et al. (48)	BNP pg/ml	-	RM 15138	14,287 (94) 13,987 (92)	5601 (37) 4,284 (28)	11,877 (78)	50	27 (14)	1,826	Device explant or deactivation	*X*^2^ test	742 (377-1090) vs. 825 (412-1565)* *p =* 0.10
Imamura et al. (47)	BNP- Log pg/ml	1	RS 27	27 (100) 22 (81)	0 (0) 27 (100)	21 (78)	35 ± 14	0 (0)	183	LVRR (EF ≥35%)	Unpaired *t*-test	2.8 ± 0.2 vs. 2.8 ± 0.3 *p =* 0.882
Imamura et al. (49)	BNP- Log pg/ml	1	R- 60	60 (100) 34 (57)	0 (0) 60 (100)	48 (80.0)	40.1 ± 12	0 (0)	183	LVRR (EF ≥35%) or device explant	Unpaired *t*-test	2.92 ± 0.30 vs. 2.89 ± 0.36 *p =* 0.793
Mano et al. (50)	BNP pg/ml	–	RS 41	41 (100) 0 (0)	0 (0) 41 (100)	28 (68)	30.1 ± 10	0 (0)	365	Device explant	Unpaired *t*-test	1140 ± 660 vs. 1282 ± 1074 *p =* 0.76

*Age in years is described in Mean ± SD.*

*Median (IRQ).

*BNP, B-type natriuretic peptide; BTT, bridge to transplant; CF, continuous flow; CI confidence interval; DT, destination therapy; FU, follow up; HR, hazards ratio; IQR, inter quartile range; ICM, ischemic cardiomyopathy; Log, log transformed, LVAD, left ventricular assist device; LVRR, left ventricular reverse remodeling; M, multicenter; NP, natriuretic peptide; NT-proBNP; N-terminal pro-B-type NP; N, number; OR, odds ratio; R, retrospective cohort; S, single center; SD, standard deviation; vs., versus.*

*The bold p-values indicate statistical significance*.

#### All-Cause Mortality

Five studies analyzed the predictive value of NPs for all-cause mortality, of which 4 studies included BNP and 1 study included NT-proBNP (Table 2). None of the studies found BNP levels before LVAD implantation predictive of all-cause mortality (28–31). In contrast, NT-proBNP levels before LVAD implantation were predictive of 30-days all-cause mortality (32). The study by Cabiati et al. looked at an associative relation and found that NT-proBNP was associated with 4-weeks all-cause mortality, while NT-proANP and NT-proCNP were not (33).

#### Right Ventricular Failure

Four studies assessed the predictive value of NPs for RVF (two studies BNP, one study proBNP, and one study NT-proBNP) (Table 3) (34–37). All studies had at least the outcome “need for right ventricular assist device (RVAD).” Study sample size was 54–79, with the exception of the study by Loghmanpour et al. with a large study population of *N* = 10,909 (34–37). The two studies analyzing BNP demonstrated that BNP levels before LVAD implantation were predictive of the need of RVAD postoperatively up to 14 days (34, 35). In the study of Shiga et al. it was demonstrated that BNP levels ≥1,200 pg/ml were not predictive of RVF, while in the study by Kato et al. BNP levels ≥1,232 ng/ml were an independent predictor of RVF after 2–14 days (34, 35). In a Bayesian prediction model, proBNP levels had high predictive value for RVF in 2–14 days after LVAD implantation (36). NT-proBNP levels before LVAD implantation were not predictive of RVF within 48 hours post-operatively (37). Importantly, this study by Potapov et al. used a cut-off value of NT-proBNP >10,000 pg/ml (37). Of the seven studies analyzing an associative relation, four analyzed BNP, one analyzed proBNP, and two analyzed NT-proBNP (34, 35, 37–41). Shiga et al. found that BNP levels were associated with “need for RVAD” within the postoperative period (34). Both Hennig et al. and Potapov et al. found that NT-proBNP was associated with RVF within 48 hours postoperatively (37, 41). The remaining studies did not find an association between NP levels and RVF, although in some studies results were close to statistical significance (35, 38–40).

#### Major Adverse Events

A total of five studies assessed the predictive or associative relation between NPs and MAEs. The following MAEs were identified in these studies: VA, AR, “combined adverse events” (including episode of VA, HF, chest pain, bleeding, infection, thrombosis, pump-related problems, biliary disfunction, elective procedures), complicated postoperative stay, and all-cause rehospitalization. Three studies analyzed whether NPs were predictive of MAEs, of which two studies assessed BNP and one study assessed NT-proBNP (Table 4). All studies found that BNP and NT-proBNP levels before LVAD implantation were predictive of MAEs (42–44). Hellman et al. demonstrated that BNP was an independent predictor for VA within 15 days post-operative (43). In a large study by Truby et al. BNP >500 ng/l was predictive of the development of moderate or severe AR (42). NT-proBNP measured at “hospital admission” before LVAD implantation was an independent predictor for rehospitalization due to cardiac, bleeding, infection, thrombosis, pump related, biliary, or “elective” events (44). Of the studies reporting on associative relations, three studies analyzed BNP levels and two studies analyzed NT-proBNP levels (42, 44–46). In these studies, BNP levels before LVAD implantation were associated with MAEs between 2 weeks up to 2 years (42, 43, 45). In a sub-analysis, Hegarova et al. demonstrated that although BNP was associated with adverse events up to 1.5 years after initial discharge, it was not associated with subsequent rehospitalizations (45). The two studies analyzing NT-proBNP levels found that it was not associated with complicated post-operative stay. However, it was associated with less rehospitalization for combined adverse events (44, 46).

#### Myocardial Recovery

Only one study assessed the predictive value of NP for myocardial recovery (Table 5). This study found that BNP levels before LVAD implantation were not predictive of LV recovery after 6 months (47). Five studies reported on associative relations between NP and myocardial recovery. All studies analyzed BNP levels, whereas Topkara et al. additionally investigated proBNP levels. Besides the large study by Topkara et al. none of the included studies found an association between BNP and LV recovery (6, 47–50).

## Discussion

To the best of our knowledge, this is the first systematic review assessing the prognostic value of circulating NP levels in advanced HF patients receiving LVAD therapy. The main findings are as follows:

1. B-type natriuretic peptide is not predictive of all-cause mortality at a follow-up of 3 months or longer. Evidence regarding NT-proBNP is insufficient to draw a reliable conclusion.
2. B-type natriuretic peptide is predictive of RVF in the postoperative period after the first 48 hours. In contrast, NT-proBNP seems associated with RVF within 48 hours after LVAD implantation.
3. B-type natriuretic peptide and NT-proBNP levels appear to be predictive of various MAEs, and related to rehospitalization up to 1.5 years after LVAD implantation.
4. B-type natriuretic peptide is not predictive of, and most likely not associated with, myocardial recovery.

### All-Cause Mortality

None of the studies found that BNP levels before LVAD implantation are predictive of all-cause mortality up to 2 years after implantation. In contrast, Topilsky et al. demonstrated that preoperative NT-proBNP levels are predictive of 1-month mortality after LVAD implantation (32). The study by Papathanasiou et al. analyzing BNP, had a similar study sample size and baseline characteristics compared to the study by Topilsky et al., but did not report a significant predictive relation (28). The differences in type of NPs and length of follow-up may have contributed to this contradictory finding. The follow-up duration may be an important factor, as both studies analyzing 1-month mortality found a significant relation (32, 33). This may suggest that NPs are related to early postoperative mortality, but lose their prognostic value for all-cause mortality at longer follow-up. Of note, both studies analyzed NT-proBNP, and to date no studies are available analyzing BNP levels in relation to 1-month mortality after LVAD implantation. Whether BNP and NT-proBNP have different prognostic power regarding all-cause mortality after LVAD implantation needs to be investigated in future prospective studies.

In the studies included in this review, BNP levels are not predictive of all-cause mortality after LVAD implantation. This is an interesting finding, since NPs (including BNP and NT-proBNP) are strong predictors of all-cause mortality in HF patients (2, 20–22). In addition, BNP levels are independent predictors of mortality in advanced HF patients receiving cardiac resynchronization defibrillator (CRT-D) therapy (51, 52). It may well be that NPs are not so much a predictor of mortality risk after LVAD implantation, but rather a reflection of disease severity. Furthermore, the change in prognostic value of NPs may be caused by several mechanisms related to the LVAD itself. The device unloads the LV, thereby reducing pressure and stretch of cardiomyocytes. Reduced myocardial stretch may lead to lower NP levels. Decreased NP levels are related to a lower mortality risk (53). In parallel with the improvements in hemodynamics and prognosis provided by the LVAD, NT-proBNP levels decrease after LVAD implantation (27, 49). However, they remain abnormal and elevated compared to the levels in chronic HF patients, suggesting that key pathological changes on cellular myocardial level remain, despite LVAD support (49). This may partly be explained by the fact that the flow mechanisms of the devices, including lack of pulsatility and high rotation speed of the LVAD disc or propeller, influence several physiological processes connected to NPs, like neurohormonal changes and sympathetic and renin-angiotensin-aldosterone activity (27, 49, 51). These processes may result in altered NP release. Therefore, the prognostic value of NP levels before LVAD implantation may be changed by the therapy itself. Nonetheless, several studies have shown that NP measurements and their fluctuations during LVAD therapy are strongly related to adverse outcome including mortality (28, 29, 45). These findings may suggest, that NP levels before implantation and during LVAD support may not have similar predictive value for all-cause mortality, as the hemodynamic support provided by the LVAD may change NP levels and the accompanied mortality risk.

Finally, another explanation for the lack of predictive value of NPs regarding all-cause mortality could be the erratic course of LVAD therapy. Major adverse event rates are high and correspond to high mortality rates. The studies included in this review that found no relation between NPs and all-cause mortality had a follow-up duration of 90, 180, and 730 days after LVAD implantation. Competing risk analyses should have been performed to account for the effect of MAEs on mortality. However, none of the studies included in this review provided these analyses. This statistic error could explain why in the included studies NPs are not predictive for mortality, but appear to have predictive value for various MAEs.

### Right Ventricular Failure

Four studies investigated whether NP levels were predictive of RVF after LVAD implantation. Due to the large study by Loghmanpour et al. the sample size of the studies (BNP, proBNP) reporting a positive predictive value for RVF was 11.049 patients, whereas the total sample size of the studies (NT-proBNP) which reported no predictive value was 54 patients (34–37). All studies analyzing BNP and proBNP were predictive, whereas the study by Potapov et al. analyzing NT-proBNP was not. It should be noted that this is only one study with a small study population (37). Nevertheless, this finding may be linked to the type of NPs that was investigated. In addition, this could be explained by the follow-up duration. Potapov et al. investigated RVF within 48 hours postoperatively, whereas all other studies analyzed RVF after the first 48 hours post LVAD implantation (34–37). Furthermore, it should be noted that in all studies, the definition of RVF included “need for RVAD.” Since the decision to use an RVAD after LVAD implantation may vary based on clinical practice, this may change the definition of outcome and thereby the prognostic value of NPs for prediction of RVF.

It is well-known that RVF after LVAD implantation severely impairs prognosis. In the INTERMACS registry, RVF represented the specific cause of death in 4% of all patients (4). The interaction between the LVAD, the right ventricle (RV), and NP system is complex. Preoperative elevated NP levels, inflammatory markers and cytokines may represent a worse hemodynamic status and therefore a higher susceptibility to RVF after LVAD implantation (41). At the same time, it has been suggested that elevations in neurohumoral markers and cytokines may directly influence RV function, contributing to the development of RVF (41).

One study included in our review analyzed late RVF after LVAD implantation, and found no relation of BNP levels before LVAD implantation to late RVF (mean follow-up of 3.4 years) (38). This may be explained by the fact that development of RVF during long-term support is most likely multi-factorial. Different from the LV, the RV does not exhibit significant reverse structural remodeling despite reduced RV afterload during LVAD support (54–56). Kato et al. demonstrated that the CF-LVAD impairs the physiological contractility of cardiomyocytes by the non-pulsatile mode of LV unloading, which over time could lead to decreased RV compliance and contractility (40, 57). Furthermore, interventricular septum displacement caused by suction of the CF-LVAD may result in RV dysynchrony and also reduced cooptation of the tricuspid valve. In long-term LVAD therapy, this may gradually increase tricuspid regurgitation and subsequent increase RV preload. Over time these factors could contribute to the development of late RVF (38). Future studies should address preoperative circulating NP levels in relation to these different factors, in order to better predict RVF during long-term LVAD support.

### Major Adverse Events

In the INTERMACS registry, the most frequently reported MAEs after LVAD implantation are infection, neurologic events, RVF, device malfunction including pump thrombosis, and multiple system organ failure (4). However, apart from RVF, there were no studies available that assessed the relation between NPs and these specific MAEs. The studies included in this review assessed the relation between NP levels before LVAD implantation and MAEs including VA, AR, “combined adverse events,” complicated postoperative stay, and all-cause rehospitalization. All studies that were included found that NP (BNP and NT-proBNP) levels before LVAD implantation are predictive of diverse MAEs occurring in the postoperative period within 15 days up to 2 years follow-up, and rehospitalizations within 1.5 year after LVAD implantation (42–44).

#### Ventricular Arrhythmia

Hellman et al. demonstrated that high BNP levels before LVAD implantation are a powerful predictor for VA up to 15 days (43). Several mechanisms may explain this finding. BNP levels reflect ventricular stretch and hypertrophy, which over time results in tissue fibrosis and other changes of the myocardium that may be a substrate for VA (58). The LVAD unloads the failing heart, but cannot initiate reverse remodeling within 15 days. Thus, the substrate for VA remains, as does the prognostic value of BNP before LVAD implantation. Another possible explanation may be that high BNP levels are associated with elevated levels of cytokines and catecholamines, resulting in prolongation of the action potential and enhanced calcium entry, causing QTc prolongation and promoting arrhythmogenesis, eventually triggering VA (43, 59).

#### Aortic Regurgitation

During CF-LVAD therapy, up to 15% of the patients may develop moderate to severe AR, with significant impact on morbidity and mortality (42). The study by Truby et al. identified BNP levels >500 ng/L as a predictor in a univariate analysis of a cox proportional hazard model for the development of moderate or severe AR after 2 years of LVAD therapy. However, BNP levels were not taken into account in the multivariate analysis of AR development (42). Factors like body mass index, sex, and destination therapy strategy appear to be stronger predictors of development of moderate or severe AR than BNP levels (42). Nevertheless, this study points out that BNP identifies patients who are vulnerable for adverse events. In patients with AR, NPs are predictive of the development of HF and mortality (60). However, no studies are available that assess the prognostic value of NPs in relation to AR in (advanced) HF patients. Therefore, more studies are needed to get mechanistic insights into the relation between NPs and AR in HF patients receiving LVAD therapy.

#### Rehospitalization and Combined Adverse Events

Two studies analyzed rehospitalization after initial discharge, of which Hasin et al. found higher NT-proBNP before LVAD implantation predictive for, and associated with, less rehospitalization for any MAE (44). Hegarova et al. demonstrated that higher BNP levels were associated with less or no combined adverse events that required outpatient care or rehospitalization. In a sub-analysis, the authors found that BNP levels were not able to differentiate between combined adverse events that required rehospitalization and those that did not (45). Interestingly, both studies demonstrated that higher NP levels before LVAD implantation were related to less combined adverse events (44, 45). This finding may be related to the kind of MAE. Hasin et al. found that cardiac events (30.4%) including VA, HF, and chest pain, and bleeding events (29.6%) were the main reasons for rehospitalization, whereas Hegarova et al. found that pump thrombosis (29%) and decompensated HF (26%) were the most frequent adverse events (44, 45). These findings suggest that NP levels before LVAD implantation within a certain range may be predictive for rehospitalization of specific MAEs.

### Myocardial Recovery

Among the articles considered in this systematic review, five studies investigated the relation of NPs with myocardial recovery. Imamura et al. demonstrated that BNP before LVAD implantation was not predictive for LV ejection fraction recovery (47). The study by Topkara et al. demonstrated that NP levels (BNP, proBNP) were associated with myocardial recovery, while the other studies did not (6, 47–50). The total sample size in the four studies that found no association was just slightly larger than the sample size of the one study that did, mainly due to the large studies by Topkara et al. (6) and Wever-Pinzon et al. (48). Both authors extracted their data from the INTERMACS registry, had comparable inclusion criteria, baseline characteristics, outcome and median follow-up. Nevertheless, they found conflicting results. This may be related to the fact that Wever-Pinzon et al. additionally included patients implanted with pulsatile-flow LVADs, and a relatively high number of INTERMACS level 1 patients, who were in critical cardiogenic shock at time of device implantation (48). The higher number of INTERMACS level 1 patients may explain the higher levels of BNP found within the recovery group, and may diminish the associative relation between lower levels of BNP and myocardial recovery. Taken together, these reports are indicative for the fact that NPs may not be a specific marker for cardiac recovery, but rather reflect the general physical condition and the severity of HF in advanced HF patients receiving LVAD therapy.

### Limitations

Although we systematically assessed the evidence for NP and its role as prognostic biomarker in advanced HF patients who receive LVAD therapy, our study is not devoid of its own limitations. According to the NOS score, most studies included in our review were of good quality. However, a number of these studies had a small patient population and therefore low statistical power. The heterogenous nature of the data in terms of timing of NP measurements, subtypes of NP, follow-up time, statistical analyses, and end-points pre-empted us from performing a meta-analysis and derive definitive conclusions. In addition, in a number of studies included in this review, the predictive value of NPs in LVAD patients was not the main hypothesis. We were not able to asses all end-points because of limited literature, and several end-points had heterogenous and subjective definitions, such as “need for RVAD” for the definition of RVF. Although it is generally accepted that NP levels are influenced by gender, age, BMI, comorbidities, kidney disease, and HF medication, most studies did not take all variables into account. In addition, some bias was created as the manuscripts from the same authors, and several studies analyzing multiple subtypes of NP or end-points, were included.

### Future Perspective

Given the high incidence of MAEs after LVAD implantation, optimization of patient selection is crucial in order to improve outcome after LVAD implantation. Circulating NP levels may have some power predicting MAEs, RVF and rehospitalization during LVAD therapy. However, new, more promising, circulating biomarkers have been identified for prognostication of MAEs and mortality in HF patients (15, 61–64). Multi-biomarker panels seem to improve the prognostic power of these biomarkers. Emdin et al. compared a multi-biomarker panel [NT-proBNP, soluble suppression of tumorigenicity-2 (sST2), high-sensitive troponin T (hs-TnT)] with a single biomarker (NT-proBNP). Relative risk for all-cause mortality was higher among patients with elevated levels of all multi-panel biomarkers compared to patients with elevated levels of a single biomarker (NT-proBNP, sST2, hs-TnT; RR 9.5 vs. NT-proBNP; RR 2.3, respectively) (65). Ahmad et al. showed that novel biomarkers, such as galectin-3 (GAL-3), ST2, growth differentiation factor-15 (GDF-15), high sensitive C-reactive protein (hs-CRP), and copeptin, when stratified by baseline NT-proBNP levels in their cohort of advanced HF patients, were more sensitive of maladaptive processes than traditional laboratory markers with established prognostic significance, such as red blood cell distribution width, creatinine, blood urea nitrogen, and sodium, which remained within normal limits (66). These studies show that novel biomarkers and their multi-biomarker panels may reflect disease severity more accurately than currently used metrics (65–67). In addition, these novel biomarkers provide a unique insight into the pathophysiologic changes of HF as they reflect the different maladaptive processes involved e.g., oxidative stress, fibrosis, and inflammation (68). Therefore, novel biomarkers may be considered for screening of patients with advanced HF requiring CF-LVAD therapy, and monitoring of LVAD patients. The present systematic review demonstrates that in order to improve generalizability and interpretation, large prospective studies with predefined outcome, and follow-up duration analyzing preimplantation NPs, multi-biomarker panels and their changes over time are warranted. Validated assays in consecutive patients should be used, and detailed cardiovascular profiles should be created to systematically define pathologies contributing to the levels of NP and other circulating biomarkers.

## Conclusions

This systematic review demonstrates that BNP levels before LVAD implantation are not predictive of all-cause mortality after LVAD implantation. The implantation of an LVAD appears to alter prognosis and NP levels to such an extent that prognosis for mortality stratified by NP levels before LVAD implantation is not applicable after LVAD implantation. However, NP levels appear to identify advanced HF patients who are at risk for postoperative RVF and MAEs, such VA, AR, and rehospitalization. More studies regarding the timing of NP measurements, using different subtypes of NPs within prospective cohorts with predetermined end-points and follow-up are needed to confirm the prognostic value of NPs in advanced HF patients who will receive LVAD therapy.

## Data Availability Statement

The original contributions presented in the study are included in the article/Supplementary Material, further inquiries can be directed to the corresponding author.

## Author Contributions

EJ: conceptualization: lead, formal analysis: lead, investigation: equal, methodology: equal, project administration: lead, software: equal, writing–original draft: lead, writing–review & editing: equal. JJ: conceptualization: lead, investigation: equal, methodology: equal, supervision: lead, writing- original draft: equal, writing–review & editing: equal. SB: conceptualization: lead, methodology: equal, supervision: equal, writing–original draft: equal, writing–review & editing: equal. MS: conceptualization: equal, supervision: equal, writing- original draft: equal, writing–review & editing: equal. LT: conceptualization: lead, formal analysis: equal, methodology: equal, supervision: lead, writing- original draft: lead, writing–review & editing: lead. All authors contributed to the article and approved the submitted version.

## Conflict of Interest

The authors declare that the research was conducted in the absence of any commercial or financial relationships that could be construed as a potential conflict of interest.
