# Supplementary material for: Prognostic Value of Natriuretic Peptides for All-Cause Mortality, Right Ventricular Failure, Major Adverse Events, and Myocardial Recovery in Advanced Heart Failure Patients Receiving a Left Ventricular Assist Device: A Systematic Review
Source: Front Cardiovasc Med. 2021 Jul 7;8:699492. doi: 10.3389/fcvm.2021.699492 (PMC8292668; doi:10.3389/fcvm.2021.699492)
Supplement: Supplementary file 2 [file Data_Sheet_2.docx]

|  | Supplementary Material Supplement 2: Newcastle-Ottawa Quality Assessment Form for Cohort Studies **Assessment of quality of a cohort study Newcastle–Ottawa Scale** | | | | | | | | | | |
| --- | --- | --- | --- | --- | --- | --- | --- | --- | --- | --- | --- |
|  | | Observational cohort studies | | | | | | | | | |
|  | | Papathanasiou (28) | Sato  (29) | Yoshioka (30) | Shiga (31) | Topilsky (32) | Cabiati (33) | Shiga (34) | Kato (35) | Loghmanpour (36) | Potapov (37) |
| **Selection** | | | | | | | | | | | |
| 1) Representativeness of the exposed cohort  a*. Truly representative*  b*. Somewhat representative*  c. *Selected group*  d. *No description of the derivation of the cohort* | |  |  |  |  |  |  |  |  |  |  |
| 2) Selection of the non-exposed cohort  a*. Drawn from the same community as the exposed cohort*  b. *Drawn from a different source*  c*. No description of the derivation of the non-exposed cohort* | |  |  |  |  |  |  |  |  |  |  |
| 3) Ascertainment of exposure  a*. Secure record (e.g., surgical record)*  b*. Structured interview*  *c. Written self-report*  d. *No description*  e. *Other* | |  |  |  |  |  |  |  |  |  |  |
| 4) Demonstration that outcome of interest was not present at start of study  a. *Yes*  b. *No* | |  |  |  |  |  |  |  |  |  |  |
| **Comparability** | | | | | | | | | | | |
| 1) Comparability of cohorts on the basis of the design or analysis controlled for confounders  a. *The study controls for age, sex and marital status*  b. *Study controls for other factors (list)*  c. *Cohorts are not comparable on the basis of the design or analysis controlled for confounders* | |  |  |  |  |  |  |  |  |  |  |
| **Outcome** | | | | | | | | | | | |
| 1) Assessment of outcome  a. *Independent blind assessment*  b. *Record linkage*  c. *Self-report*  d. *No description*  e. *Other* | |  |  |  |  |  |  |  |  |  |  |
| 2) Was follow-up long enough for outcomes to occur  a. *Yes*  b. *No* | |  |  |  |  |  |  |  |  |  |  |
| 3) Adequacy of follow-up of cohorts  a. *Complete follow up; all subject accounted for*  b. *Subjects lost to follow up unlikely to introduce bias; number lost less than or equal to 20% or description of those lost suggested no different from those followed*  c. *Follow up rate less than 80% and no description of those lost*  d. *No statement* | |  |  |  |  |  |  |  |  |  |  |
| **Summary** | | | | | | | | | | | |
| **Selection**  **Comparability**  **Outcome**  **Total** | | **7** | **7** | **7** | **6** | **7** | **7** | **7** | **7** | **7** | **7** |
| **Thresholds for converting the Newcastle-Ottawa scales to AHRQ standards (good, fair, and poor):**  ***Good quality:*** *3 or 4 stars in selection domain AND 1 or 2 stars in comparability domain AND 2 or 3 stars in outcome/exposure domain*  ***Fair quality:*** *2 stars in selection domain AND 1 or 2 stars in comparability domain AND 2 or 3 stars in outcome/exposure domain*  ***Poor quality:*** *0 or 1 star in selection domain OR 0 stars in comparability domain OR 0 or 1 stars in outcome/exposure domain* | | **GOOD** | **GOOD** | **GOOD** | **GOOD** | **GOOD** | **GOOD** | **GOOD** | **GOOD** | **GOOD** | **GOOD** |

| Observational cohort studies | | | | | | | | | | | | | |
| --- | --- | --- | --- | --- | --- | --- | --- | --- | --- | --- | --- | --- | --- |
| **Selection** | | | | | | | | | | | | | |
| Deswarte (39) | Kapelios (38) | Pettinari (40) | Hennig (41) | Hellman (43) | Hegarova (45) | Truby  (42) | Hasin (44) | Hasin (46) | Imamura (47) | Topkara (6) | Wever-Pinzon (48) | Imamura (49) | Mano  (50) |
|  |  |  |  |  |  |  |  |  |  |  |  |  |  |
|  |  |  |  |  |  |  |  |  |  |  |  |  |  |
|  |  |  |  |  |  |  |  |  |  |  |  |  |  |
|  |  |  |  |  |  |  |  |  |  |  |  |  |  |
| **Comparability** | | | | | | | | | | | | | |
|  |  |  |  |  |  |  |  |  |  |  |  |  |  |
| **Outcome** | | | | | | | | | | | | | |
|  |  |  |  |  |  |  |  |  |  |  |  |  |  |
|  |  |  |  |  |  |  |  |  |  |  |  |  |  |
|  |  |  |  |  |  |  |  |  |  |  |  |  |  |
| **Summary** | | | | | | | | | | | | | |
| **7** | **7** | **7** | **7** | **7** | **7** | **7** | **8** | **8** | **7** | **7** | **7** | **7** | **6** |
| **GOOD** | **GOOD** | **GOOD** | **GOOD** | **GOOD** | **GOOD** | **GOOD** | **GOOD** | **GOOD** | **GOOD** | **GOOD** | **GOOD** | **GOOD** | **GOOD** |
